# Serum estradiol (E2), progesterone (P), and human chorionic gonadotropin (HCG), D-dimer (D-D) fibrinogen (FIB) levels after low molecular weight heparin sodium on preventing miscarriage in patients with adverse pregnancy and delivery

**DOI:** 10.5937/jomb0-56635

**Published:** 2025-09-05

**Authors:** Liucheng Pei, Ting Wang, Qiuling Yang

**Affiliations:** 1 Wuhai Maternal and Child Health Hospital, Department of Gynecology, Wuhai, 016000, Inner Mongolia Autonomous Region, China

**Keywords:** serum estradiol (E2), progesterone (P), human chorionic gonadotropin (HCG), D-dimer (D-D), fibrinogen (FIB), LMWH sodium, adverse pregnancy, tocolysis, pre-thrombotic state (PTS), serumski estradiol (E2), progesteron (P), humani horionski gonadotropin (HCG), D-dimer (D-D), fibrinogen (FIB), natrijum-niskomolekularni heparin (LMWH), rizična trudnoća, tokoliza, pretrombotično stanje (PTS)

## Abstract

**Background:**

This study investigates the effects of subcutaneous injection of low molecular weight heparin (LMWH) sodium on preventing miscarriage in patients with adverse pregnancy and delivery. Specifically, it evaluates changes in serum estradiol (E2), progesterone (P), and human chorionic gonadotropin (HCG), as well as D-dimer (D-D) and fibrinogen (FIB) levels following treatment.

**Methods:**

A total of 82 patients with adverse pregnancy admitted to Wuhai Maternal and Child Health Hospital from April to December 2024 were randomly assigned to either the control group (CG, n=41) receiving dydroges-terone therapy or the observation group (OG, n=41), which received LMWH sodium in addition to dydroges-terone. The study assessed changes in sex hormone levels, pre-thrombotic state (PTS) indices, thromboelastogram (TEG) parameters, and overall treatment outcomes before and after treatment.

**Results:**

Following treatment, E2, P, and HCG levels were significantly higher in the OG compared to the CG (P<0.05). Additionally, coagulation markers such as activated partial thromboplastin time (APTT), prothrombin time (PT), thrombin time (TT), and plasminogen (PLG) were significantly elevated in the OG, while FIB and D-D levels were lower compared to the CG (P<0.05). TEG analysis showed that the OG exhibited higher R and K values, whereas MA, Angle, and CI values were lower than in the CG (P<0.05). The total response rate in the OG (95.12%) was significantly higher than in the CG (75.61%) (P<0.05). Additionally, the OG had lower rates of neonatal malformation, low birth weight, and mortality compared to the CG, with significant differences in malformation and low birth weight rates (P<0.05).

**Conclusions:**

Subcutaneous injection of LMWH sodium effectively improves sex hormone levels, reduces the risk of PTS, and enhances pregnancy outcomes in patients with adverse pregnancy and delivery. The therapy also improves coagulation and fibrinolytic markers, leading to better maternal and neonatal outcomes.

## Abstract (sr)

**Uvod:**

Ova studija istražuje efekte subkutane injekcije natrijum-niskomolekularnog heparina (LMWH) u prevenciji pobačaja kod pacijentkinja sa rizičnom trudnoćom i porođajem. Konkretno, procenjuju se promene u nivoima serumskog estradiola (E2), progesterona (P) i humanog horionskog gonadotropina (HCG), kao i nivoi D-dimera (D-D) i fibrinogena (FIB) nakon tretmana.

**Metode:**

Ukupno 82 pacijentkinje sa rizičnom trudnoćom su primljene u Bolnicu za zdravlje majki i dece Wuhai od aprila do decembra 2024. godine i nasumično raspoređene u kontrolnu grupu (CG, n=41), koja je primala terapiju didrogesteronom, ili posmatranu grupu (OG, n=41), koja je uz didrogesteron dodatno primala natrijum-niskomolekularni heparin. Studija je procenjivala promene u nivoima polnih hormona, indeksima pretrombotičnog stanja (PTS), parametrima tromboelastograma (TEG) i ukupnim ishodima lečenja pre i nakon tretmana.

**Rezultati:**

Nakon tretmana, nivoi E2, P i HCG bili su značajno viši u OG u poređenju sa CG (P<0,05). Pored toga, markeri koagulacije, poput aktiviranog parcijalnog trombo-plastinskog vremena (APTT), protrombinskog vremena (PT), trombinskog vremena (TT) i plazminogena (PLG), bili su značajno povišeni u OG, dok su nivoi FIB i D-D bili niži u poređenju sa CG (P<0,05). TEG analiza je pokazala da je OG imala više vrednosti R i K, dok su MA, Ugao i CI vrednosti bile niže nego u CG (P<0,05). Ukupna stopa odgovora u OG (95,12%) bila je značajno viša nego u CG (75,61%) (P<0,05). Pored toga, OG je imala nižu stopu neonatalnih malformacija, niske porođajne mase i mortaliteta nego CG, s tim da su razlike u stopama malformacija i niske porođajne mase bile značajne (P<0,05).

**Zaključak:**

Supkutana injekcija natrijum-niskomolekularnog heparina efikasno poboljšava nivoe polnih hormona, smanjuje rizik od PTS i unapređuje ishode trudnoće kod pacijentkinja sa rizičnom trudnoćom i porođajem. Terapija takođe poboljšava koagulacione i fibrinolitičke markere, poboljšavajući materinske i neonatalne ishode.

## Introduction

Adverse pregnancy and childbirth refer to a series of complications that occur during pregnancy or childbirth. Pregnant women who experience adverse pregnancy and childbirth may face physical pain, emotional distress, and psychological pressure. On the other hand, the fetus faces health risks, which may lead to growth restriction and even endanger the life of the fetus in severe cases. Common adverse pregnancies include miscarriage, preterm birth, gestational hypertension, preeclampsia, fetal malformation, placental insufficiency, intrauterine growth restriction (IUGR), and other conditions. Recent studies have shown that obstetric disorders, such as recurrent spontaneous abortion (RSA), threatened miscarriage, IUGR, intrahepatic cholestasis of pregnancy (ICP), and preeclampsia. The pre-thrombotic state (PTS) and thrombosis mechanism in the mother’s important organs or uterus and placenta [1]. PTS refers to a pathological hypercoagulable state of the blood in the human body, which is characterized by the disruption of the balance of various molecular, cellular, and biochemical processes among the coagulation system, fibrinolytic system, and anticoagulant system in the body. The pathological changes are often caused by genetic or acquired defects in anticoagulant proteins, coagulation factors, and fibrinolytic proteins, leading to further occurrence of various haematological changes in the body that promote thrombosis [2]. If a woman has abnormal vascular endothelial cell function, abnormal balance of blood components, abnormal platelet number and function, abnormal hemorheological system, and abnormal coagulation, anticoagulation, and fibrinolysis systems during pregnancy, it may cause pathological hypercoagulable state, which is manifested as increased adhesion of platelets to the vessel wall, excessive activation of the coagulation process, and decreased thrombolytic ability. Then, PTS is formed and eventually develops into thrombosis [3]. The presence of PTS increases thrombosis risk in pregnant women. It reduces blood flow velocity at the maternal-placental junction [4], which leads to thrombosis and increases the risk of adverse pregnancy and childbirth history, such as pregnancy-related hypertension syndrome and embryo suspension [5].

In clinical practice, a variety of means, such as uterine protection treatment, drug treatment, surgical treatment, and maintenance treatment, are often used for adverse pregnancy and childbirth [6]. Because the structure of dydrogesterone is very similar to progestins (such as progesterone), it can mimic progestins and play a role in maintaining normal pregnancy. In some cases of adverse pregnancy, the level of progesterone may decrease, resulting in reduced ability to support pregnancy and then problems such as threatened abortion. Dydrogesterone can partially replace the progesterone function, protect the embryo by regulating the endometrium and reducing uterine contraction, and help maintain the stability of pregnancy. Therefore, dydrogesterone is commonly used in the clinical treatment of adverse pregnancy reactions such as threatened abortion. However, the effect of dydrogesterone is not obvious for patients with severe conditions. Low molecular weight heparin (LMWH) prevents thrombosis, stimulates angiogenesis, and increases vascular permeability [7] [8]. It also inhibits the proliferation of vascular smooth muscle and helps to increase placental blood perfusion in the fetus. It can also inhibit vascular smooth muscle proliferation and help increase placental perfusion to fetal blood, thereby improving perinatal outcomes [9] [10]. As against unfractionated heparin, LMWH has high bioavailability, long anticoagulant effect, few complications, and will not produce teratology and other toxic side effects on the fetus through the placenta [11] [12], and is not secreted in breast milk. Currently, relevant studies have investigated the use of LMWH in pregnant women [13] [14] [15]. LMWH sodium is a commonly used anticoagulant drug in clinical practice. Therefore, this article explored the application effect of subcutaneous injection of LMWH sodium on preventing miscarriage in patients with adverse pregnancy and pregnancy based on routine clinical treatment.

## Materials and methods

### Subjects

Eighty-two patients with adverse pregnancy and delivery admitted to Wuhai Maternal and Child Health Hospital from April 2024 to December 2024 were enrolled and rolled into the observation group (OG) and control group (CG) using a random number table, each with 41 cases. The general data of patients in two groups were compared, including a series of data such as age, gestational age, gravidity, parity, and body mass index (BMI). The results suggested that the data of the two groups were comparable. The ethics committee of Wuhai Maternal and Child Health Hospital approved the trial.

### Inclusion and exclusion criteria

Before treatment, informed consent was signed. The specific inclusion criteria are given in Table 1. Table 2

**Table 1:** Inclusion criteria.

Serial number	Inclusion criteria
1	In accordance with the relevant diagnostic criteria of adverse pregnancy and childbirth
2	Those who had not received any tocolysis medication during pregnancy
3	People with normal coagulation function
4	Singleton pregnancy
5	The male’s semen examination was normal

**Table 2:** Exclusion criteria.

Serial number	Exclusion criteria
1	Patients with cardiovascular, cerebrovascular, liver, kidney, and other serious diseases
2	Infection with genital tract viruses or other pathogens
3	People with habitual abortion
4	Those with an ectopic pregnancy or hydatidiform mole
5	People with severe mental disorders
6	People who are allergic to drugs

The specific exclusion criteria are presented in Table 1.

### Treatment methodologies

Patients in CG were treated with bed rest, reduced activity, and basic therapy such as oral folic acid and vitamin E, together with oral dydrogesterone (Duphaston, Abbott Biologicals B.V. Company, batch number: H20170221, size: 10 mg). The initial dose of treatment was 40 mg/d, and it was changed to 10mg twice daily after 1 day of oral administration, with an interval of 8 h. On this basis, OG was given subcutaneous injection of LMWH sodium (Jilin Huakang Pharmaceutical Co., LTD., H20010233, standard: 0.5 mL: 5,000U), once a day, 5,000U each time. Both groups were treated with dydrogesteroneuntil the progesterone test results were normal and vaginal bleeding stopped. On this basis, OG adopted subcutaneous injection of LMWH sodium for one week. If hemorrhagic adverse reactions occur, the drug should be stopped immediately.

### Observation indicators

### (1) Sex hormone levels

All patients took 8 mL of blood samples from veins before and after treatment and fasted for at least 8 hours before blood collection. The samples were centrifuged at 4,500 r/min for 10 min. The levels of estradiol (E2), progesterone (P), and human chorionic gonadotropin (HCG) in the samples were measured with the chemiluminescence immunoassay (Roche, model E601) and the supporting electrochemiluminescence kit.

### (2) PTS evaluation

Coagulation system: Blood samples were obtained for routine coagulation tests, including activated partial thromboplastin time (APTT), thrombin time (TT), prothrombin time (PT), and fibrinogen (FIB) levels.

Fibrinolysis system: blood samples were obtained by the above methods, D-dimer (D-D) was detected by URIT-600 automatic coagulation analyzer, and plasminogen (PLG) was obtained by chromogenic substrate method.

Thromboelastogram (TEG): 5 mL of venous blood samples were collected from subjects in two groups and mixed with 0.2 mL of 109 mmol/L sodium citrate solution in vacuum anticoagulant tubes. The mixture was thoroughly mixed and analyzed using a Thromboelastogram instrument (Model 5000, manufactured by Weimei (Shanghai) Management Co., Ltd.) along with corresponding quality control materials and reagents to calculate R-value, K value, Angle value, MA value, and CI value.

### (3) Evaluation of application effect

*Clinical Disease Diagnosis and Efficacy Evaluation Standards* to evaluate the treatment outcome: Cure: After 12 h of treatment, the clinical symptoms of adverse pregnancy and childbirth disappeared, and there was no abdominal or lower abdominal discomfort, vaginal bleeding, and high body temperature. At the end of the treatment, the patients were confirmed to be normal pregnancies by B ultrasound and gynaecological examination. Effective: after 24 h of treatment, the clinical symptoms disappeared, abdominal or lower abdominal discomfort was relieved, vaginal bleeding was reduced or stopped, and the body temperature tended to be normal. At the end of the treatment, B ultrasound and gynaecological examination were performed to confirm the normal pregnancy. No effect: following therapy, clinical symptoms were not improved, and the patient still had abdominal or lower abdominal pain, vaginal bleeding and high body temperature. Abnormal pregnancy was diagnosed by B ultrasound and gynaecological examination. Total effective cases = (cured case number + effective casenumber)/total casenumber×100%. The data of newborns in two groups were tracked and recorded, including malformation, premature birth, death, low birth weight, and neonatal Apgar score.

### Statistical methods

Data were analyzed using SPSS 20.0 software. Continuous variables were expressed as mean ± standard deviation (x̄±s) and were tested for normality using the Kolmogorov-Smirnov test. If normally distributed, comparisons between two groups were performed using the independent sample t-test; for nonnormally distributed data, the Mann-Whitney U test was applied. Categorical variables were presented as frequencies (n) and percentages (%) and were compared using the Chi-square test (χ^2^ test). When expected frequencies in any cell were less than 5, the Fisher’s exact test was used. A P-value of <0.05 was considered statistically significant.

## Results

### Comparison of general data of patients

Patients’ age, gestational age, parity, and BMI were collected through physical examination, medical records review, and a questionnaire survey. In CG, the average age was (27.87±4.01) years old, ranging from 24–42, with a mean gestational age of (8.17±2.04) weeks; the number of pregnancies was 1–3 (1.67±0.62); the number of deliveries was 1–3 (2.75±0.24); and the average BMI was (26.6±3.1). The mean age of OG was (28.63±2.34), ranging from 23 to 44. The average gestational age was (8.21±2.11) weeks; the number of pregnancies was 0–3 (2.52±0.37); the number of deliveries was 0–4 (2.52±0.37); and the average BMI was (27.4±3.2) (Table 3). The results showed similar general data between the two groups (P>0.05), and the two groups were comparable.

**Table 3:** Basic information of patients.

	Age	Gestational age	Number of pregnancies	Number of deliveries	BMI
CG (n=41)	27.87±4.01	8.17±2.04	1.67±0.62	2.65±0.25	26.6±3.1
OG (n=41)	28.32±3.55	8.21±2.11	1.58±0.33	2.52±0.37	27.4±3.2
t /χ^2^	-0.545	-0.327	0.347	0.292	-0.631
P	0.736	0.775	0.628	0.876	0.651

### Sex hormone levels

**Figure 1:**
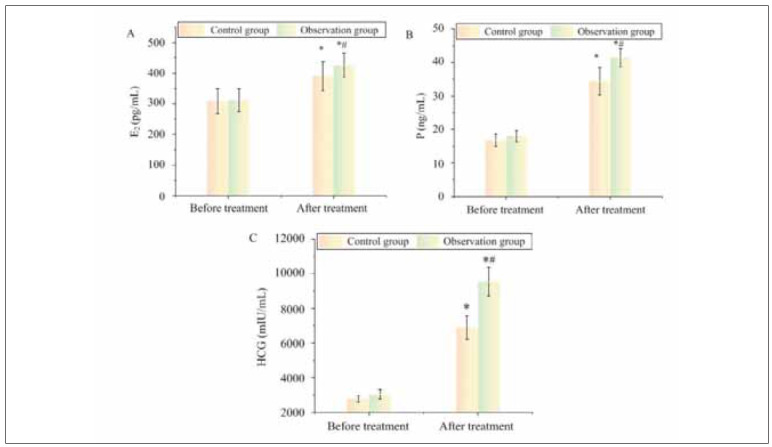
Comparison of sex hormone levels.<br>Note: * As against pre-therapy, P<0.05; #P<0.05 as against CG

Through treatment, the E2, P, and HCG levels in CG were 391.08±47.13 pg/mL, 34.41±4.11 ng/mL, and 6,893.22±666.32 mlU/mL, respectively. Those in OG were 426.32±39.92 pg/mL, 41.36±2.79 ng/mL, and 9,530.41±812.43 mlU/mL, respectively. Before treatment, E2, P, and HCG levels were similar in the two groups (P>0.05). As against before therapy, E2, P, and HCG in two groups post-treatment were raised in different ranges (P<0.05). Among them, the levels of sex hormones in OG post-treatment were superior as against CG post-therapy (P<0.05) (Figure 1).

### Coagulation system indicators

**Figure 2:**
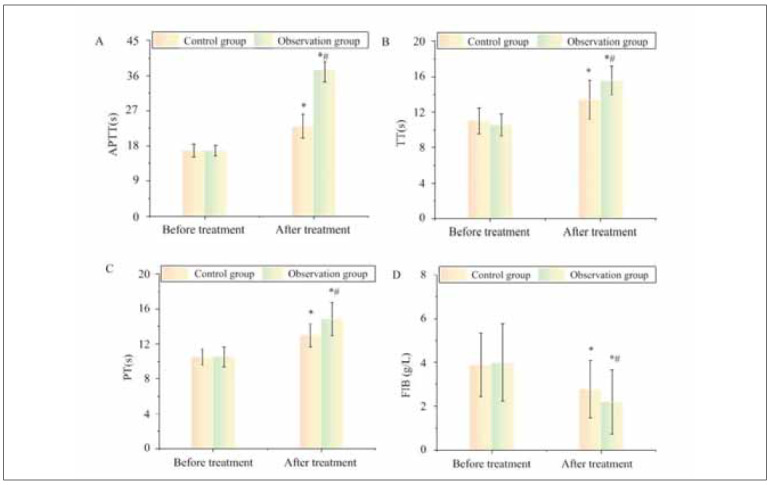
Comparison of coagulation system indexes.<br>Note: * As against pre-therapy, P<0.05; #P<0.05 as against CG

Following treatment, APTT, TT, PT were 23.02±3.04s, 13.41±2.19s, 12.97±1.30s, FIB was 2.17±1.31g/L in CG; Those in OG were 37.41±3.04s, 15.57±1.63s, 14.83±1.88s, and 2.19±1.47g/L. Before treatment, APTT, TT, PT, and FIB differed slightly between the two groups (P>0.05). Following treatment, APTT, TT, and PT were higher, and FIB was lower than pre-therapy (P<0.05). Compared with CG, the levels of APTT, TT, PT, and FIB in OG raised or decreased more clearly post-treatment (P<0.05) (Figure 2).

### Fibrinolytic system indexes

**Figure 3:**
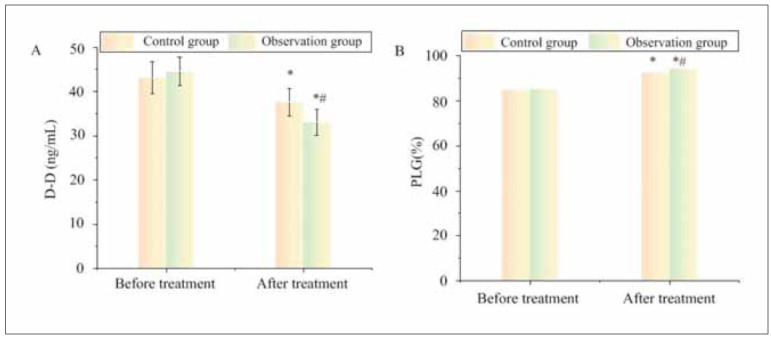
Comparison of fibrinolytic system indexes.<br>Note: * As against pre-therapy, P<0.05; #P<0.05 as against CG

Following treatment, D-D and PLG in CG were 37.63±3.11 ng/mL and 92.8%, respectively; those in OG were 33.08±2.97 ng/mL and 95.34%, respectively. The two groups had similar D-D and PLG levels pre-therapy (P>0.05). As against pre-therapy, the D-D levels were decreased, and PLG levels were raised in two groups following treatment (P<0.05). The changes of each index in OG following treatment were more significant than against CG (P<0.05) (Figure 3).

### TEG parameters

**Figure 4:**
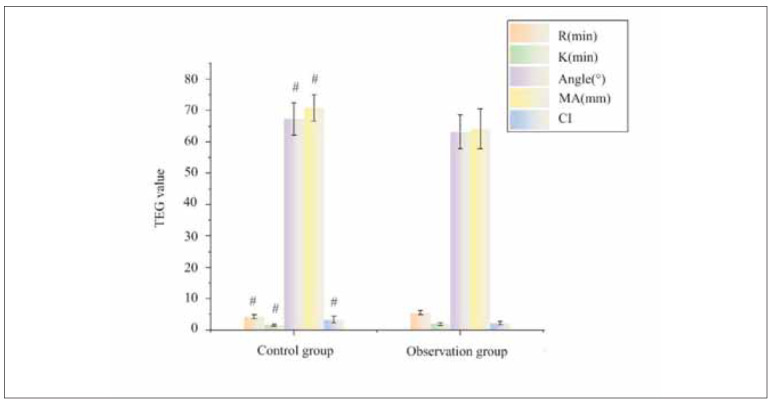
Comparison of coagulation system indexes.<br>Note: * As against pre-therapy, P<0.05; #P<0.05 as against CG

As shown in Figure 4, post-treatment, the Rvalue, K value, Angle, MA value, and CI value for the CG were 4.24±0.59 min, 1.56±0.38 min, 67.32± 5.14°, 70.88±4.23 min, and 3.32±1.07, respectively. For the OG, these values were 5.48±0.67 min, 1.91±0.46 min, 63.19±5.36°, 64.19±6.42 min, and 2.16±0.58. It can be observed from the figure that post-treatment, patients in the OG had higher R and K values compared to the CG, while MA, Angle, and CI values were lower than those in the CG, with all differences being statistically significant (P<0.05).

### Application effect evaluation

### (1) Treatment outcome of patients

In CG, 19 cases were cured, 12 were compelling, and 10 were ineffective, with a total response rate of 75.61%. In OG, 25 cases were cured, 14 were effective, and 2 were ineffective, with a total response rate of 95.12%. The percentage bar chart of treatment outcomes is given in Table 4. The total effective rate of OG was higher than CG ( 2=5.23, P<0.05).

**Table 4:** Comparison of treatment outcomes.

	Cured	Effective	Ineffective	Total effective rate
OG (n=41)	25	14	2	95.12%
CG (n=41)	19	12	10	75.61%
χ^2^				5.23
*P*				0.037

### (2) Neonatal conditions

Based on successful treatment, the neonatal condition of the adverse pregnancy and delivery patients in each group was counted (Table 5). The number of newborns with malformation, low weight, and death in OG was lower relative to CG, and the difference in the number of newborns with malformation and low weight between groups was statistically meaningful (P<0.05). Apgar score <7 and the number of newborns with death differed inconsiderably between groups (P>0.05).

**Table 5:** Neonatal conditions. Note: * denotes Fisher’s exact test.

	n	Deformity of body	Low weight	Apgar score <7	Death
CG	31	2 (6.45)	14 (45.16)	2 (6.45)	1 (3.23)
OG	39	0 (0)	3 (7.69)	2 (5.13)	0 (0)
χ^2^		4.907	8.753	-	-
*P*		0.012	0.046	>0.05*	>0.05*

## Discussion

Adverse pregnancy and childbirth remain significant concerns in maternal-fetal medicine, contributing to increased morbidity and mortality. These complications encompass a broad spectrum of conditions, including miscarriage, preterm birth, stillbirth, fetal malformations, intrauterine growth restriction (IUGR), and placental insufficiency [16] [17]. The pathophysiology of adverse pregnancy outcomes is complex and often linked to maternal coagulation disorders, endothelial dysfunction, and inflammatory processes that compromise placental function [18]. Interventions targeting these underlying mechanisms are essential for improving pregnancy outcomes.

The present study assessed the efficacy of low molecular weight heparin (LMWH) sodium combined with dydrogesterone in preventing miscarriage and improving maternal and neonatal health in patients with adverse pregnancy and delivery. Our findings indicate that LMWH sodium significantly improved pregnancy outcomes by enhancing sex hormone levels, reducing the prothrombotic state (PTS), and improving coagulation and fibrinolysis balance. The total response rate in the LMWH-treated group (95.12%) was significantly higher than in the control group (75.61%) (P<0.05), reinforcing its effectiveness as an adjunct therapy. These results align with previous studies demonstrating that LMWH administration improves pregnancy outcomes in high-risk patients, particularly those with thrombophilia, recurrent pregnancy loss (RPL), or preeclampsia [19] [20]. LMWH exerts anticoagulant, anti-inflammatory, and angiogenic effects, which collectively contribute to improved placental perfusion, reduced fetal growth restriction, and enhanced maternal vascular health [21]. Compared to traditional dydrogesterone therapy alone, LMWH sodium appears to provide additional protective effects by reducing coagulation abnormalities and increasing the likelihood of a successful pregnancy.

Hormonal balance is crucial for successful implantation, placental development, and pregnancy maintenance. Our study found that estradiol (E2), progesterone (P), and human chorionic gonadotropin (HCG) levels were significantly higher in the LMWH-treated group compared to the control group after treatment (P<0.05). These findings suggest that LMWH sodium positively influences hormonal support during pregnancy, which may contribute to reduced miscarriage risk. Estradiol promotes endometrial proliferation and vascularization, ensuring a favorable environment for fetal development. Progesterone stabilizes the uterine lining, suppresses contractions, and prevents immune rejection of the embryo, while HCG maintains corpus luteum function and stimulates progesterone production to sustain early pregnancy [22]. These findings are consistentwith previous studies that have linked LMWH ntherapy with increased progesterone levels and improved pregnancy outcomes in women with a history of recurrent miscarriage [23]. The mechanism behind this hormonal enhancement is not entirely understood but may involve LMWH-mediated improvements in placental function and reduced inflammatory responses, leading to better hormone secretion and fetal support.

Pregnancy is a prothrombotic state characterized by increased coagulation factors, reduced fibrinolysis, and endothelial dysfunction. These physiological changes are exacerbated in patients with adverse pregnancy outcomes, leading to placental thrombosis, ischemia, and fetal growth restriction [24]. Our study demonstrated that LMWH therapy significantly altered coagulation markers, as reflected in increased APTT, PT, and TT levels, indicating a prolonged coagulation time and reduced thrombotic tendency. Additionally, fibrinogen (FIB) and D-dimer (D-D) levels decreased post-treatment, suggesting improved fibrinolysis and lower clot burden, while elevated plasminogen (PLG) levels enhanced the body’s ability to dissolve clots and maintain placental circulation. These findings align with prior studies showing that LMWH reduces maternal hypercoagulability and enhances placental blood flow, thereby improving pregnancy outcomes [25]. Given that abnormal coagulation and fibrinolysis are major contributors to pregnancy complications such as preeclampsia, intrauterine growth restriction, and miscarriage, the observed improvements in coagulation markers support the use of LMWH in high-risk pregnancies.

Thromboelastography (TEG) provides a comprehensive assessment of coagulation status by evaluating clot initiation, strength, and dissolution. Our study found that patients in the LMWH-treated group exhibited higher R and K values, indicating a delay in clot initiation and reduced clot formation tendency, while lower MA and Angle values suggested reduced clot firmness and decreased hypercoagulability. These findings support the hypothesis that LMWH sodium mitigates the hypercoagulable state often seen in patients with adverse pregnancy outcomes. Similar studies have reported that LMWH therapy significantly improves TEG parameters in women with a history of thrombosis or recurrent pregnancy loss [26]. The ability of LMWH to modulate thrombotic risk and improve placental circulation may explain the better neonatal outcomes observed in our study.

In addition to improving maternal health, LMWH sodium was associated with better neonataloutcomes. The incidence of neonatal malformation and low birth weight was significantly lower in the LMWH-treated group (P<0.05), suggesting that improved placental perfusion and reduced thrombosis contributed to better fetal growth. While some studies have reported mixed results regarding LMWH’s impact on neonatal outcomes, our findings support its role in reducing fetal complications associated with placental dysfunction [15].

Despite the encouraging findings, our study has several limitations. The sample size was relatively small, which may limit the generalizability of the results, and larger multicenter trials are needed for further validation. Long-term neonatal outcomes, such as cognitive development and metabolic health, were not assessed in this study and should be explored in future research. Additionally, while LMWH sodium showed significant benefits in reducing PTS and miscarriage risk, the study did not investigate its effectiveness in specific subpopulations, such as women with autoimmune disorders or thrombophilias, where the response to anticoagulant therapy may vary. The multiplicity of analyses performed increases the risk of type I errors, and future studies should employ stricter statistical corrections to validate these findings.

## Conclusion

In summary, subcutaneous LMWH sodium administration improved sex hormone levels, enhanced coagulation and fibrinolysis balance, and contributed to better pregnancy and neonatal outcomes in patients with adverse pregnancy and delivery. The therapy effectively reduced the prothrombotic state, likely contributing to improved placental function and fetal growth. While our results support LMWH sodium as a valuable adjunct to conventional dydrogesterone therapy, further large-scale studies are needed to confirm these benefits and explore long-term maternal and neonatal outcomes.

## Dodatak

### Acknowledgements

The authors sincerely thank the staff and medical personnel of Wuhai Maternal and Child Health Hospital for their assistance in patient recruitment and data collection. We also express our gratitude to the participating patients and their families for their cooperation throughout the study.

### Funding

This study received the funding from Medical and Health Innovation Research Project of Wuhai City.

## Author contributions

Liucheng Pei: Conceptualization, methodology, formal analysis, writing—original draft.

Ting Wang: Data curation, statistical analysis, validation, writing—review & editing.

Qiuling Yang: Supervision, project administration, funding acquisition, manuscript revision.

All authors have read and approved the final manuscript.

### Ethics approval and consent to participate

This study was approved by the Ethics Committee of Wuhai Maternal and Child Health Hospital. All participants provided written informed consent before enrollment, and the study was conducted in accordance with the Declaration of Helsinki.

### Data availability statement

The datasets generated and analyzed during the current study are available from the corresponding author upon reasonable request.

### Consent for publication

Not applicable.

### Conflict of interest statement

All the authors declare that they have no conflict of interest in this work.
